# Mitochondrial genome sequencing and analysis of scuticociliates (*Uronema marinum*) isolated from *Takifugu rubripes*

**DOI:** 10.1080/23802359.2018.1483757

**Published:** 2018-07-03

**Authors:** Ruijun Li, Yanqi Gao, Yulin Hou, Shigen Ye, Lianshun Wang, Jingxian Sun, Qiang Li

**Affiliations:** a Agriculture Department Key Laboratory of Mariculture & Stock Enhancement in North China's Sea, Dalian Key Laboratory of Marine Animal Disease Control and Prevention, Dalian Ocean University, Dalian, China;; b Department of Ocean Technology, College of Marine and Biology Engineering, Yancheng Institute of Technology, Yancheng, China

**Keywords:** *Takifugu rubripes*, scuticociliates, *Uronema marinum*, mitogenomics

## Abstract

Scuticociliates are dangerous parasitic pathogens for in worldwide mariculture. Scuticociliates cause high mortality to marine fish. After an outbreak of scuticociliatosis in *Takifugu rubripes* in Liaoning Province, northern China, *Uronema marinum*, a scuticociliate, was identified. In this study, using Illumina MiSeq next-generation sequencing, the mitochondrial genome of *U. marinum* was assembled and analysed phylogenetically using mitochondrial genomes of other scuticociliates. The complete *U. marinum* mitochondrial genome was 39,845 bp; it contained two rRNAs, six tRNAs, and 39 protein-coding genes (PCGs). From the 39 PCGs, 15 PCGs were located on the heavy strand, and 24 PCGs on the light strand of *U. marinum* mitogenome. The phylogenetic tree showed that there were two main clades, Oligohymenophorea and Spirotrichea. Nine ciliate species were clustered together within Oligohymenophorea; *Uronema marinum* was a separate cluster sharing a relatively close ancestry with Hymenostomatida. The results of this study will help advance the systematics, and studies of evolution and molecular epidemiology of scuticociliates.

*Uronema marinum* is a scuticocilaite that belongs to order Shieldophilus, family Sphingidae, and genus *Uronema*. It causes scuticociliatosis, a disease with a high mortality in marine fish (Yoshinaga and Nakazoe, 1993; Sterud et al. 2000; Jee et al. 2001; Anderson et al. 2009). Recently, after an outbreak of severe scuticociliatosis in *Takifugu rubripes* in factory farming in Liaoning Province, northern China (38.9728 N and 121.3326 E), we isolated a pure pathogenic ciliate from the skin ulcers of ill *T. rubripes* and identified it as the scuticociliate *U. marinum*. Although there are many scuticociliate species reported, such as *Pseudocohnilembus persalinus* (Kim et al. 2004), *Phiasterides dicentrachi* (Sungmi et al. 2004), and *Miamiensis avidus* (Jung et al. 2005), the information of Scuticociliatida mitochondrial genome is very limited. Moreover, the mt genome sequence of *U. marinum* was still in the blank.

In this study, the total DNA of *U. marinum* was extracted, and stored in Dalian Key Laboratory of Marine Animal Disease Control and Prevention, Dalian Ocean University, Dalian, China. The mitochondrial genome of *U. marinum* was assembled using Illumina MiSeq Next-generation sequencing by SC Gene Company (Guangzhou, China). The sequence of *U. marinum* mitochondrial genome was deposited in GenBank (accession number MG272262). The whole mitogenome of *U. marinum* was 39,845 bp. Its nucleotide composition was as follows: A, 39.83%; T, 41.17%; C, 8.94%; and G, 10.06%. There were two rRNAs, six tRNAs, and 39 protein-coding genes (PCGs). Furthermore, the genes were coded on both the heavy strand and the light strand. From the 39 PCGs of *U. marinum* mitogenome, 15 PCGs were located on the heavy strand, and 24 PCGs on the light strand. Six genes (*cox1*, *nad1_a*, *orf346*, *rpl2*, *ymf65*, *cob*) used the start codon ATT. Seventeen genes (*rpl14*, *ymf70*, *nad4*, *rps3*, *nad10*, *nad7*, *rps14*, *rpl6*, *ymf75*, *nad1_b*, *atp9*, rpl16, *nad3*, *nad9*, *cox2*, *ymf56*, *ymf68*) used the start codon ATG. Eight genes (*ymf57*, *rps13*, *nad2*, *yejR*, *orf202*, *nad4l*, *nad5*, *ymf67*) used the start codon ATA. Two genes (*nad6*, *rps19*) used the start codon GTG. Four genes (*orf141*, *rps12*, *ymf64*, *ymf63*) used the start codon TTA. The genes *orf149* and *ymf66* used the start codon TTG and ATC, respectively. The stop codon TAA was present in 38 PCGs. Only the gene *ymf56* used the stop codon TAG. The G + C content of PCGs was 18.4%, very close to the 19% average G + C content of *U. marinum* mitogenome. The overall number of codon occurrences was 10,345. The most frequent amino-acid encoding codons were Leu (13.71%), Phe (11.69%), Asn (9.98%), Ile (9.73%), Lys (8.08%), Tyr (7.51%), Ser (6.83%), and Val (5.43%). Two rRNA genes (*rnl,* 2,566 bp; and *rns* 1,509 bp) were located on the heavy strand of *U. marinum* mitogenome. The G + C content of rRNA genes was 23.38% for *rnl*, and 24.78% for *rns*, which was higher than the average 19% G + C content of the *U. marinum* mitogenome. There were six tRNAs on the mitogenome of *U. marinum*. *tRNA-Glu*, *tRNA-Met*, and *tRNA-Trp* were on the heavy strand, and *tRNA-Tyr*, *tRNA-Phe*, and *tRNA-His* were on the light strand. These six tRNA genes had the predicted typical cloverleaf secondary structure.

Phylogenetic relationships between 12 ciliates in the Oligohymenophorea taxa were analysed according to the nucleotide and amino-acid sequences of 13 PCGs from GenBank using BI and ML methods. The resulting phylogenetic tree showed that there were two main clades, Oligohymenophorea and Spirotrichea (Figure 1). Nine ciliate species were clustered together within Oligohymenophorea; *Uronema marinum* was a separate cluster, sharing a relatively close ancestry with Hymenostomatida.

**Figure 1.**
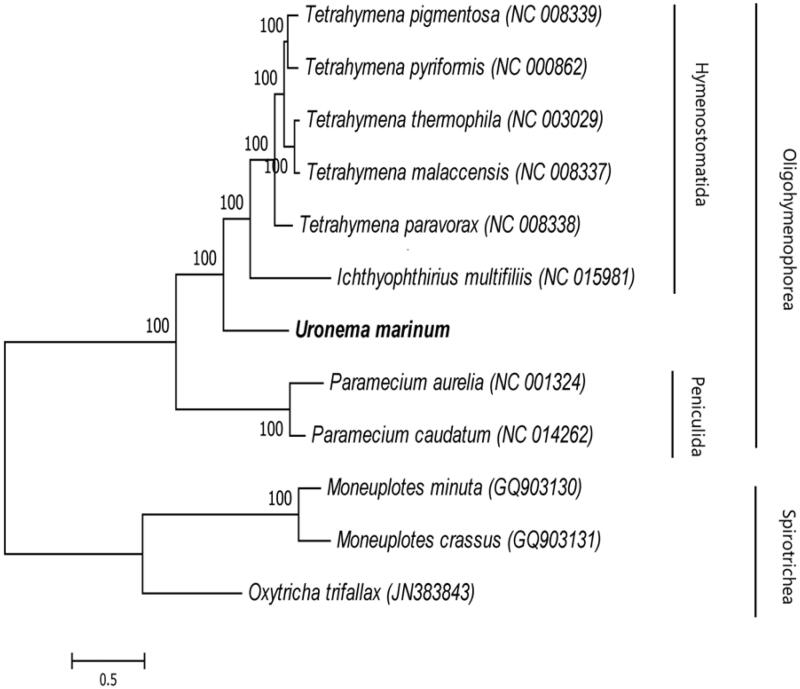
Phylogeny of *Uronema marinum*. Phylogenetic tree inferred from the amino-acid sequences predicted in the mitogenome of other ciliates. The number of the branches indicates posterior probabilities (BI).
